# The identification of requirements for competency development during work-integrated learning in healthcare education

**DOI:** 10.1186/s12909-024-05428-9

**Published:** 2024-04-22

**Authors:** Oona Janssens, Vasiliki Andreou, Mieke Embo, Martin Valcke, Olivia De Ruyck, Marieke Robbrecht, Leen Haerens

**Affiliations:** 1 Department of Educational Studies, Faculty of Psychology and Educational Sciences, Ghent University, H. Dunantlaan 2, Ghent, 9000 Belgium; 2 Department of Movement and Sports Sciences, Faculty of Medicine and Health Sciences, Ghent University, Ghent, 9000 Belgium; 3 Department of Public Health and Primacy Care, Academic Center for General Practice, KU Leuven, Kapucijnenvoer 7, Leuven, 3000 Belgium; 4 Expertise Network Health and Care, Artevelde University of Applied Sciences, Voetweg 66, Ghent, 9000 Belgium; 5 Imec-mict-UGent, Miriam Makebaplein 1, Ghent, 9000 Belgium; 6 Department of Industrial Systems Engineering and Product Design, Faculty of Engineering and Architecture, Ghent University, Campus Kortrijk, Graaf Karel de Goedelaan 5, Kortrijk, 8500 Belgium; 7 Department of Communication Sciences, Ghent University, Campus Ufo Vakgroep Communicatiewetenschappen Technicum, T1, Sint‑Pietersnieuwstraat 41, Ghent, 9000 Belgium; 8 Department of Internal Medicine and Pediatrics, Faculty of Medicine and Health Sciences, Ghent University, C. Heymanslaan 10, Ghent, 9000 Belgium

**Keywords:** Continuous professional development, Competency-based education – continuous competency development, Healthcare education, Multidisciplinary research, Work-integrated learning, ePortfolio

## Abstract

**Background:**

Work-integrated learning (WIL) is widely accepted and necessary to attain the essential competencies healthcare students need at their future workplaces. Yet, competency-based education (CBE) remains complex. There often is a focus on daily practice during WIL. Hereby, continuous competency development is at stake. Moreover, the fact that competencies need to continuously develop is often neglected.

**Objectives:**

To ultimately contribute to the optimization of CBE in healthcare education, this study aimed at examining how competency development during WIL in healthcare education could be optimized, before and after graduation.

**Methods:**

Fourteen semi-structured interviews with 16 experts in competency development and WIL were carried out. Eight healthcare disciplines were included namely associate degree nursing, audiology, family medicine, nursing (bachelor), occupational therapy, podiatry, pediatrics, and speech therapy. Moreover, two independent experts outside the healthcare domain were included to broaden the perspectives on competency development. A qualitative research approach was used based on an inductive thematic analysis using Nvivo12© where ‘in vivo’ codes were clustered as sub-themes and themes.

**Results:**

The analysis revealed eight types of requirements for effective and continuous competency development, namely requirements in the context of (1) competency frameworks, (2) reflection and feedback, (3) assessment, (4) the continuity of competency development, (5) mentor involvement, (6) ePortfolios, (7) competency development visualizations, and (8) competency development after graduation. It was noteworthy that certain requirements were fulfilled in one educational program whereas they were absent in another. This emphasizes the large differences in how competence-based education is taking shape in different educational programs and internship contexts. Nevertheless, all educational programs seemed to recognize the importance of ongoing competency development.

**Conclusion:**

The results of this study indicate that identifying and meeting the requirements for effective and continuous competency development is essential to optimize competency development during practice in healthcare education.

**Supplementary Information:**

The online version contains supplementary material available at 10.1186/s12909-024-05428-9.

## Background

*“During an internship, you have to guide the student at the competence level he/she* ***is positioned at***. *At the end of the internship, you have to assess the student at the competence level he/she* ***should be positioned at***.*” (R14 - associate degree nursing)*.

Work-integrated learning plays a vital role in healthcare education. WIL can be seen an umbrella term for a range of approaches and strategies that integrate theory with healthcare practice supported by a well-defined curriculum [1]. Students report manifold positive outcomes of learning in real-life contexts [2]. It allows students to familiarize with the external demands from the healthcare system, regulatory organizations, and the societal expectations towards healthcare professionals. While the benefits of WIL are widely recognized, its implementation remains complex. To address this, competency-based education (CBE) has been introduced as a supportive approach for WIL processes [3].

CBE emphasizes the integration of knowledge, skills, and attitudes in a specific context and shifts the focus from time-based training (hours of curriculum representation) to outcome-oriented competencies. Students are stimulated to attain competencies, and these can be acquired throughout the full educational continuum instead of during a limited time window or internship [3, 4]. CBE is defined as *‘an approach to prepare healthcare professionals for practice that is fundamentally oriented to graduate outcome abilities and organized around competencies derived from an analysis of societal and patient needs. It de-emphasizes time-based training and promises greater accountability, flexibility, and learner-centredness’* [4]. Specific and generic competencies (e.g., communication, collaboration) are both essential to become a comprehensive healthcare professional [5]. These two types of competencies can be seen as a part of the T-shaped professional where specific competencies include disciplinary knowledge while generic competencies are the encompassing professional abilities that allow someone with profound disciplinary knowledge to interact meaningfully with others. These competencies are transferable to other healthcare disciplines [6]. However, both types of competencies need to be attained during a student’s educational career.

 Continuous competency development is crucial to provide qualitative patient care, and various well-known competency frameworks have been established to guide this competency development; e.g., the Canadian Medical Education Directives for Specialists (CanMEDS) [7], the Tomorrow’s Doctors [8, 9], the Scottish Doctor [10], and the Accreditation Council for Graduate Medical Education (ACGME) [11]. A competency framework consists of different levels of competencies where the highest level contains one or more lower levels that concretize the higher level(s). For example, the CanMEDS competency framework consists of seven Roles (= highest level; e.g., Expert). Each role contains different key competencies (= second level; e.g., practice medicine within their defined scope of practice and expertise) in turn consisting of several enabling competencies (= third and lowest level; e.g., demonstrate a commitment to high-quality care of their patients) [7]. Enabling competencies mostly are further subdivided into assessment criteria that are concrete enough to allow competency assessment.

Competency frameworks ensure consistent standards across settings and provide standardized assessments of competency development [12, 13]. Next to the competency levels of a competency framework, some educational programs incorporate levels of proficiency or performance within their competency framework to support learners’ competency development e.g., beginner, intermediate and advanced. These levels indicate how well a student has mastered a particular competency. This enables a comprehensive description and scaffolding of students’ competency development.

Next to the addition of levels of proficiency or performance to a competency framework to optimize continuity by emphasizing progress, the supplementation of Entrustable Professional Activities (EPA) to the competency framework might offer opportunities to bridge the theory-practice gap. EPAs are introduced to structure healthcare educational programs emphasizing the autonomy and global competence level of a student instead of the assessment of separate competencies. A matrix might be constructed to align the developed EPAs with the already existing competencies to meaningfully describe the desired outcomes of trainees. The EPA approach makes use of levels of entrustment (e.g., trusted to observe only; trusted to execute with direct supervision and coaching; trusted to execute with indirect supervision and discussion of information conveyed for most simple and some complex cases; trusted to execute with indirect supervision and may require discussion of information conveyed but only for selected complex cases; trusted to execute without supervision) to describe different levels that students move through during their learning process starting from the predefined lowest level and moving to the predefined highest level [14, 15]. This continuous development dimension is considered key to implement CBE in healthcare education [16].

However, learners often neglect continuous competency development, focusing solely on their daily practices. This results in a fragmentation of learning in practice hindering the continuity of WIL [17]. Nevertheless, competency development *within* an internship, and also *between* internships, requires that competency development is not a static collection of separate competencies but a dynamic and continuous process that extends beyond graduation into healthcare professionals’ entire careers [17–19].

Although there is a limited amount of literature in healthcare education that thoroughly describes competency development and its implementation, the literature suggests that an ePortfolio can be used to measure and monitor competency development [20]. It might offer advantages in improving the transition from the educational institution to work, and in supporting competency development after graduation by optimizing continuous professional development (CPD) [1, 21–26]. Nevertheless, the value of ePortfolios is only useful when ePortfolios and the herein-implemented competency development are thoroughly integrated into the student’s entire educational career and a holistic picture of competency development is being adopted [27, 28].

There is a lack of evidence describing how to attain and optimize continuous competency development before and after graduation [29]. Because the fragmented picture of learners’ competency development hinders their progress [30], this study aimed to examine how a student’s competency development can be optimized during WIL in healthcare education, before and after graduation, focusing on the experience of faculty.

## Methodology

### Context

This study took place within a larger interdisciplinary research program namely (source deleted for blinded review). This program aimed at designing, developing and evaluating an ePortfolio prototype to scaffold WIL in healthcare education. The current study was conducted in October and November 2022 in Flanders (Belgium).

### Design

We employed a qualitative study with 14 semi-structured interviews (one interview with three participants) with experts in CBE and WIL from seven different healthcare disciplines and one educational program with expertise in competency development and ePortfolio use namely business management. We organized the interviews online so that participation could be fostered by eliminating practical constraints (time savings, no travel time, no traffic problems, etc.). One interview was conducted with three participants as these participants could not find another suitable time for the interview (P1, P2, P3). In total, 16 participants attended the interviews.

### Inclusion criteria

A purposive sampling technique was used where participants were invited through e-mail after having received their contact information via internship coordinators of included educational programs.

Participants were selected based on the following inclusion criteria:

- Being affiliated to a specific healthcare educational program in Flanders and/or an educational program where students learn a large part of their competencies at the workplace;
- Being an expert in CBE and/or WIL and/or having experience with ePortfolio use at the workplace (at least three years of experience);
- Being involved in the education of students at the workplace.

The choice for including diverse healthcare educational program was made with a finding of a previous study in mind [17]. This study, conducted with healthcare students, mentors, and educators, highlighted the lacking uniformity of CBE approaches within different healthcare educational programs as a barrier for effective CBE implementation. Therefore, the inclusion of multiple healthcare educational programs might be seen as a starting point for a more uniform CBE implementation.

### Sample

Fourteen participants representing different educational programs were selected to participate because of their known context-specific expertise in healthcare education. Two extra participants were included because of their expertise with ePortfolios to support competency development in the context of business management. This to include broader perspectives that are possibly transferable to healthcare education (shown as ‘other*’ in Table 1). One of these participants was responsible for ePortfolio use in an educational institution providing multiple educational programs (P14); another person was a co-developer of another, independent ePorfolio tool scaffolding competency development (P15).

### Ethical considerations

The research procedure was carried out in accordance with relevant guidelines and regulations. Prior to the study, approval for the protocol was obtained from the Ethics Board of the University of Ghent (PA-2019-053). An informed consent and a privacy statement were presented to the participants before starting the interview. Informed consent and agreement with the privacy statement were obtained from all the participants for this study.

### Data collection

Based on the initial goal of this study to examine how a student’s competency development can be optimized during WIL, a semi-structured topic guide was developed consisting of 11 questions zooming in on the implementation of competencies and capturing continuous competency development during WIL (see Appendix 1). The first five questions mainly dealt with the implementation of competency frameworks in practice, while the other six questions dealt with capturing competency development. The interviews were each moderated by two researchers (see Table 1). Because all researchers were experts in the field who were highly aware of the developed topic guide and the process to be followed, they could actively be involved as interview moderators. The mean duration of the interviews was 67 minutes (see Table 1).

### Data-analysis

All interviews were audio-recorded with participants’ permission. Recordings were transcribed (using the online transcription tool via https://otranscribe.com). The qualitative analysis of the data is carried out by means of an inductive thematic analysis using Nvivo12©.

To ensure the completeness and transparency of our methodology and results, we shortly outline the process how the initial goal of the study (to examine how a student’s competency development can be optimized in terms of effectiveness and continuity) evolved throughout the study. After posing the predefined questions (see Appendix 1) to the participants, they often answered by stating ‘requirements for competency development’ or ‘things students need to optimally develop their competencies’. Therefore, the results of this study are described in terms of requirements for competency development during WIL in healthcare education.

An inductive thematic analysis was used because a data-driven analysis was chased [31, 32]. The coding process was first conducted by the main author. ‘In vivo’ codes were created for each sentence or paragraph of each interview by staying as close as possible to the words of the participants, emphasizing the inductive character of the analysis. Second, the main author scrutinized the individual codes to develop comprehensive sub-themes and themes, again staying as close as possible to the original data. As the analysis revealed, the themes and sub-themes were often related to requirements for a student’s competency development. Therefore, the themes were compiled into concepts based on different types of requirements. The wording of the requirements in turn emphasized the inductive character by adopting the words that were used by the participants.

When the analysis was finished, a second researcher (V.A.) coded two of the fourteen interviews (14%) to control coding reliability as this method is seen as a more efficient approach than double coding all data [33]. This coding process followed the same path as that of the first coder/researcher. Afterwards, the codes of both researchers were compared and discussed. In case of disparities, causes for the disagreement were searched and dissolved to reach consensus [31]. After this second coding process and the discussion that led to consensus, the developed themes and concepts were discussed within the research team. In case of unclarities or discrepant visions, a continuous and iterative discussion was used to lead to consensus. The final codebook can be found in Appendix 2.

## Results

There is a lack of evidence describing how to attain and optimize continuous competency development, before and after graduation. Therefore, this study aimed to examine how a student’s competency development during WIL in healthcare education could be optimized. The inductive analysis approach identified eight types of requirements for effective and continuous competency development [29].

Sample characteristics are reported in Table 1. Two of the participants identified themselves as male, the others as female, reflecting the real-life situation in healthcare where in 2020, more than 78% of the healthcare professionals were females (https://statbel.fgov.be/) (column two). The educational programs where participants were affiliated to were specified by their European Qualifications Framework (EQF) level to harmonize differences between educational programs in different countries (Compare Qualifications | Europass) (see column three). The function of each participant in relation to their educational program was outlined in column four. Column five presents the moderators and column six consists of the duration of each interview.

**Table 1:** Demographics

Participant	Gender	Educational program	Function	Moderators	Duration (minutes)
P1	♀	Family Medicine (EQF level seven)	Staff member	V.A. - O.D.R.	71
P2	♀	Family Medicine (EQF level seven)	Staff member	V.A. - O.D.R.	
P3	♀	Family Medicine (EQF level seven)	Program coordinator	V.A. - O.D.R.	
P4	♀	Pediatrics (EQF level seven)	Professor	V.A. - O.D.R.	54
P5	♂	Medicine (EQF level six)	Professor	V.A. - O.D.R.	61
P6	♀	Speech therapy (EQF level six)	Educator	O.J. - O.D.R.	70
P7	♀	Business management (EQF level six)	Competency coach	O.J. - O.D.R.	64
P8	♀	Family Medicine (EQF level seven)	Program coordinator	V.A. - O.D.R.	58
P9	♀	Audiology (EQF level six)	Internship coordinator	O.J. - O.D.R.	85
P10	♂	Nursing (EQF level six)	Educator – internship coordinator	M.E. - O.D.R.	77
P11	♀	Family Medicine (EQF level seven)	Educational support	V.A. - O.D.R.	60
P12	♀	Pediatrics (EQF level seven)	Professor	M.R. - O.D.R.	57
P13	♀	Business management (EQF level six)	Coordinator dual learning^a^	O.J. - O.D.R.	74
P14	♀	Nursing (EQF level five)	Educator - internship coordinator	O.J. - O.D.R.	70
P15	♀	Nursing (EQF level five)	Internship coordinator	O.J. - O.D.R.	75
P16	♀	Podiatry (EQF level six)	Internship coordinator	O.J. - O.D.R.	63

^a^Dual learning = includes learning in working environments and learning in school environments. Dual learning assumes that pupils and students not only apply what they have learned at school in the workplace, but also gain knowledge, skills and attitudes in the workplace which were not introduced in the school environment [34].

The analysis revealed eight types of requirements for effective and continuous competency development during WIL namely requirements for (1) competency frameworks, (2) reflection and feedback, (3) assessment, (4) the continuity of competency development (5) mentor involvement, (6) ePortfolios, (7) competency development visualizations, and (8) competency development after graduation. The following Table 2 outlines these requirements supplemented with the main findings for each of them. The third and fourth column outline the focus of each requirement. This focus demonstrates which requirements might improve the continuity aspect of competency development and which might improve the effectiveness of competency development, and how this can be done, based on the findings of this study. However, many requirements might improve both the continuity and effectiveness of a student’s competency development as they seem largely interconnected.

**Table 2:** Overview of the eight types of requirements and their focus on the effectiveness and/or continuity of competency development

Type of requirements	Main findings	How to improve the effectiveness of competency development?	How to improve the continuity of competency development?
**Competency frameworks**	The selection, utilization, and implementation of appropriate competency frameworks played a crucial role in effective competency development.	An appropriate and clear competency framework that fits to the context and is closely connected to the healthcare workplace might improve the effectiveness of competency development.	Using the same appropriate and clear competency framework throughout the entire educational program and thereafter might improve the continuity of competency development.
**Reflection and feedback**	Encouraging students to engage in reflective practices, also at a higher level than daily practices, and providing constructive feedback were identified as important factors in enhancing competency development.	The engagement of students in reflection, on daily practices ánd on global competency development, linked to the predefined competencies, might improve the effectiveness. Moreover, the involvement of mentors in giving appropriate feedback that supports a student’s learning process might improve the effectiveness of competency development.	Taking along reflections and related feedback from previous internships and program years might improve the continuity. Moreover, focusing on competency development next to focusing on separate competency units might improve the continuity of competency development.
**Assessment**	The design and implementation of robust assessment methods and tools were essential for evaluating competency development accurately and support its continuity.	The design and implementation of an assessment tool should consist of clear assessment criteria and should allow valid, objective, and smooth assessment to improve the effectiveness of competency development (e.g., matching the assessment scale to the context, use of a rubric, etc.).	The design and implementation of an assessment tool should allow taking into account the development of competencies. Also, taking the same assessment tool along might improve the continuity of competency development.
**The continuity of competency development**	Ensuring the continuity and progression of competency development throughout the WIL experience emerged as a critical requirement.		The educational program should allow students to take along their competency development throughout their educational program (e.g., by not closing a program year in the used ePortfolio); it should provide enough learning opportunities by offering a sufficient amount of internship places so that competencies might be continuously developed within similar contexts to improve the continuity of competency development.
**Mentor involvement**	Active involvement and guidance from mentors significantly influenced the effectiveness of competency development during WIL.	Ideally, one-to-one guidance is preferred. However, as this is often not feasible in practice, the awareness of mentors when it comes to a student’s competencies to achieve and the active provision of learning opportunities might improve the effectiveness of competency development.	
**ePortfolios**	The use of ePortfolios provided a valuable platform for documenting and supporting competency development.	An ePortfolio might support both the assessment for learning and the assessment of learning. Combining both in one tool might improve the effectiveness of competency development [119–122].	An ePortfolio might enable taking competency development throughout the entire internship, program year and thereafter hereby improving the continuity of competency development.
**Competency development visualizations**	Visual representations and tools that visually mapped competency development and progress facilitated the understanding and monitoring of competency development.	The detection of competency gaps might be facilitated by using an appropriate visualization so that learning opportunities could be developed, which in turn might improve the effectiveness of competency development.	A visualization of competency growth might improve the continuity, before and after graduation, as competencies are taken along and students are being stimulated to keep working on their competencies to improve the continuity of competency development.
**Competency development after graduation**	Recognizing the importance of ongoing competency development even after graduation was identified as an essential requirement.		Recognizing and emphasizing the importance of competency development after graduation might increase the intrinsic motivation of students and graduates to keep developing their competencies.

The following section describes each of the eight types of requirements.

### Requirements for competency frameworks

To support competency development, each stakeholder (student, educator, and mentor) needs to be aware of the competency framework being used. According to the participants, stakeholders often did not know the framework well, although they were highly aware of the importance of competency framework. There were differences between educators and workplace mentors where the first group often knew the framework well while mentors were mostly less aware of the used framework. However, some mentors knew the competency framework well because it was used for students to formulate learning goals at the start of an internship together with their mentor.

“No, not at all. I think they start to know the CanMEDS because we work with this framework for years. But our EPAs, no, not at all.” *(P1 – family medicine)*.

“Yes. Because the student needs to formulate his learning goals before the start of each internship and based on their previous internships.” *(P14 – associate degree nursing)*.

The CanMEDS competency framework was the most frequently used framework (audiology, medicine, family medicine, and pediatrics). Two educational programs (speech therapy and family medicine) used Entrustable Professional Activities (EPAs) as a supplement to their own program-specific framework.

“We created a matrix so that we can link each EPA to a competency from the CanMEDS competency framework. It is a matrix and when you click on an EPA, three or four linked competencies will pop up.” *(P5 – speech therapy)*.

The CanMEDS competency framework consists of seven Roles with 27 key competencies each consisting of several enabling competencies. The number of competencies within the used frameworks was different between the participants. Some educational programs limited it to ten competencies or EPAs (e.g., speech therapy) while other educational programs used a competency framework with 62 competencies or developed 62 EPAs (e.g., medicine).

“I think it is important how many lists you have and yes, keep it limited and make sure it remains relevant and that the added value is clear. I think that is the largest challenge.” *(R12 – pediatrics)*.

**Fig. 1:**
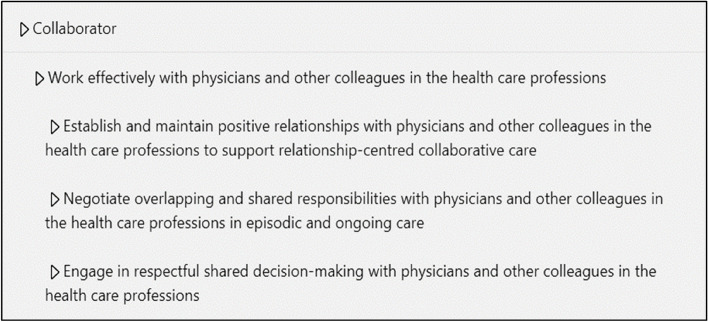
Example of an implemented three-level CanMEDS competency framework implemented in an ePortfolio

Participants stated that the number of levels within a competency framework was critical to provide a clear and workable framework. Participants believed that a balance between too many and too little levels was necessary. They said that too many levels might overcomplicate the learning process by having too much assessment criteria. In contrast, too little levels might be too vague and less clear. Most participants indicated their preference for three levels. An example of a three-level framework, implemented in an ePortfolio can be found in Fig. 1 where the highest level demonstrates the CanMEDS role, the middle level the CanMEDS key competencies, and the lowest level the CanMEDS enabling competencies.

“I think the competencies are split up too much in our educational program.” *(P12 – pediatrics)*

“We used to struggle with vague, meaningless criteria. When you have too little levels, a mentor does not know what behavior he needs to assess. But, when you become too concrete, you have a huge list where the mentor will just tick boxes without thinking.” *(R10 – nursing)*.

Participants emphasized that the competency framework needs to be adapted to the context where it will be implemented. Moreover, not only the context but also the time when it needs to be used during the educational program seems essential. It became clear that the same competency framework can be used during the complete educational program. Participants believed that the higher level competencies might remain the same while the lowest level (e.g., the assessment criteria) needs to evolve with the student’s growth.

“Throughout the modules, the competencies and assessment criteria change according to the expected growth of the student.” *(P14 – associate degree nursing)*.

“First-year students will never rate themselves as excellent. But then I say ‘yes, you are, at this moment in time, considering the expected competence level, you can be excellent.” *(P4 – pediatrics)*.

It was noticed that generic competencies (e.g., communication, collaboration, etc.) often remained the same over the course of the program, while specific competencies changed continuously over time. Moreover, the already-known problem of overexposing technical competencies was mentioned by multiple participants. The need to demonstrate all technical competencies to receive a grade enlarged this problem. According to the participants, the fact that a student could not reflect about certain competencies at a certain moment needs to be seen as a sign to create extra learning opportunities, rather than as a red flag in the context of assessment.

“It is important for a mentor to step outside the medical role and to take into account the other roles too. I think many mentors are often not aware of the fact that this is an expectation too.” *(P3 – family medicine)*.

### Requirements for reflection and feedback

Participants perceived the reflection about a student’s global competency development as problematic although this was required to support competency development.

“I think that students seldomly reflect about the bigger picture.” *(P5 – medicine)*.

Continuous, formative feedback was seen as a requirement because it gave students anchors to formulate future learning goals and to further develop their competencies.

“If you have learning goals at the start of your internship taking into account previous internships. If you then got help from a mentor who says what your previous learning goals were that you did not achieve. Then you can select them and take them with you again until you can achieve them.” *(P10 – nursing)*.

However, it seemed important to the participants that reflections and feedback were task-oriented (feedback on the performance of a task) and not person-oriented (feedback on the personality of a student).

“We ask, of course, to provide task-oriented feedback, not person-oriented feedback. What can you (not) do and what do you need? We try to pass that along to the mentor. When person-oriented feedback is given, it is not pleasant for a student that a future mentor looks at that student with preconceptions.” *(P7 – business management)*.

### Requirements for assessment

Participants believed that the assessment of competencies was indispensable. Participants stated that assessment was mostly subjective although assessment criteria were available. Giving a final score was often based on gut feeling and/or intercollegiate discussions.

“We are a pragmatic educational program. We are not that strong in educational approaches. We act from gut feeling and explore. We also discuss a lot within our internship teams.” *(P9 – associate degree nursing)*.

Furthermore, the assessment method being adopted influenced students’ competency development, according to participants. For instance, participants highly valued the nature of the scoring scale. A scale with a larger value range (e.g., a five-point scale from zero to four) could be chosen to demonstrate differences while a smaller scale range (e.g., a three-point scale from zero to two) might facilitate easy assessment (e.g. pass/fail scale). Moreover, an odd scale might stimulate scoring in the middle, especially with students. Overall, participants preferred a four-point or a five-point scale over a numeric score. Other participants preferred a pass-fail scale at the workplace as it is more logical, clear and easy-to-use.

“We would like to have ‘is a student able to do that or not?’. Our paper versions included two points: yes or no. Actually, I think it’s difficult to differentiate between ‘I can take an audiogram for 10/20 or for 7/20. No, it is sufficient or not.” *(P9 – audiology)*.

Moreover, participants believed that the addition of a rubric appeared to support assessment of competencies. A rubric is an assessment tool involving different levels of expectations to objectively assess competencies in CBE [35]. Participants perceived the opportunity of a rubric to add an explanation to each point of the scale as beneficial to clarify each point on the scale.

“But what is excellent, what is good? What does that mean for me as a student?” *(P4 – pediatrics)*.

“And with a rubric, it should be possible to make a box where you explain each assessment criterium under the higher level so that it is clear whether or not a competency is sufficient or not.” *(P10 – nursing)*.

According to participants, it seemed important that the score of the student, mentor (workplace), and educator (educational institution) matched during the assessment, although in practice this often caused problems. When scores matched, it was possible to start more in-depth reflection conversations, stated participants. Also, when a student scored below expectations, the discussion of this score with their mentor and/or educator optimized their competency development as gaps in their competency development could be detected and learning opportunities could be created based on these gaps.

“Then there is no tension. You just talk about performances, about what challenges there are… You achieve greater depth which is very valuable. The more you can let go the fixed assessment conversation, the more you can choose the topics you want to talk about in depth.” *(P10 – nursing)*.

### Requirements for the continuity of competency development

According to participants, the rigidity of the educational system, mostly regulated by governmental departments, often hinders the continuity of competency development through program years as the visibility of attained competencies and competencies to attain might differ between educational programs. In some educational programs, where participants were affiliated to, the portfolio-system closed after a program year so that outcomes of previous program years were not considered. Ideally, the system should focus on continuous competency development by making this visible during a student’s educational journey.

“But that is where our educational system makes no sense because the content of each program year needs to be closed after that program year. So, it is not that simple in our educational system but that would be the ideal image.” *(P13 – business management)*.

Most of the participants agreed that the reflections of students, and the feedback of mentors and educators of previous internships should be visible during the following internships and program years to optimally support continuous competency development. Noteworthy, other participants mentioned that the visibility of *all* the reflections and feedback was too much. They preferred only a summary because they thought students needed to start with a clean slay and every internship should be a new start.

According to all participants the same competency framework needs to be consistently used to integrate competencies throughout internships. This could facilitate the necessary transfer of competencies into practice. Ideally, students transfer what they have learned in theory (educational institution) into practice (workplace). When this was not the case, students’ competency development was hampered.

“Ideally, there is a transfer of what you learn during your education to what you do at the workplace. And these two lines should coincide. But if the line of how a student performs at the workplace systematically lies under the line of how this student performs during a more formal subject at the educational institution, then there is an indication that you have difficulties transferring competencies.” *(P5 – medicine)*.

To optimize competency development, participants thought that is important that a student was exposed sufficiently to learning opportunities. During a certain period of time it seemed interesting to see: (1) IF there were sufficient learning opportunities (e.g., students might have ‘a negative grade’ because they did not had a learning opportunity and not because they did not meet the minimum criteria), (2) HOW MANY learning opportunities a student had, and (3) HOW QUICK a student performed independently (how many learning opportunities were needed to perform independently? ).

“It can color red because you did not have the chance to perform… Then you can also interact with the workplace to create learning opportunities.” *(P13 – business management)*.

“We also have competencies where a student writes down each day what he has done during a certain internship day. In this way, we can monitor how quick a student transitions to act independently.” *(P9 – audiology)*.

Some participants stated that the possibility to provide insufficient learning opportunities might be strongly related to the limited amount of internship places complicating competency development. Furthermore, the fact that internships contexts differed when students transition from one internship to another might influence their competency development.

“For example, a student is doing his first internship in a self-employed practice where there are easy, well-defined cases. There, the expected competence level is ‘indirect supervision’. The student’s next internship takes place at the hospital where there are a lot of complex cases and the expected competence level is ‘direct supervision’. What is the growth then… Because you could say that their competence level drops.” *(P6 – speech therapy)*.

### Requirements for mentor involvement

Participants stated that the involvement of mentors might support students’ competency development. A meeting between student and mentor, and/or formulating learning goals together with the mentor before the start of an internship seemed beneficial.

“That is part of it. That is getting to know who their mentor is going to be. They invite this person and they make sure everything is prepared.” *(P9 – audiology)*.

In the same context, one-to-one guidance at the workplace was considered the best option for supervising students according to participants. This implies that a student is assigned to one single mentor. But, most participants stated this was almost impossible in practice.

“No, and certainly not now anymore. In the past, we had more fixed mentors and we could say that we had two or three mentors who guided a student during his or her internship.” *(P14 – associate degree nursing)*.

### Requirements for ePortfolios

All participants stated that an ePortfolio might support students’ competency development. They expressed specific requirements regarding the ePortfolio design to optimize competency development: (1) the possibility to archive learning artefacts, (2) the automatic input of competency frameworks, (3) the provision of an automatic score when individual competencies were assessed, (4) the easier detection of competency gaps (e.g., competencies can be visualized in one single graphical display), (5) the possibility to filter competencies based on the used competency frameworks, (6) the possibility to filter competencies based on an internship or program year, (7) the availability of the ePortfolio independent of location or time, (8) the receipt of a notification as a student when feedback was provided, (9) the receipt of a notification as a mentor or educator when feedback was needed, (10) the extraction of a curriculum vitae, (11) the upload of multimedia, (12) the different styling options (e.g., bold, cursive, etc.) that can be used to structure reflections and/or feedback, (13) the possibility to use different devices (e.g., PC, smartphone), and (14) the customizable structure and/or competency frameworks within an ePortfolio (e.g., the names of the competency levels within a competency frameworks could be adjusted).

### Requirements for competency development visualization (e.g., in an ePortfolio)

Most participants said that visualizations to support competency development were not yet frequently used. The only visualizations that were used by some of the participants were the use of colors to show the level of individual competencies. It was not possible to track the competency development as the visualizations were only used within one internship or semester. Six participants explicitly stated that visualizations of competency development highly appealed to them. Most participants did not know the opportunities that visualizations could offer.

**Fig. 2:**
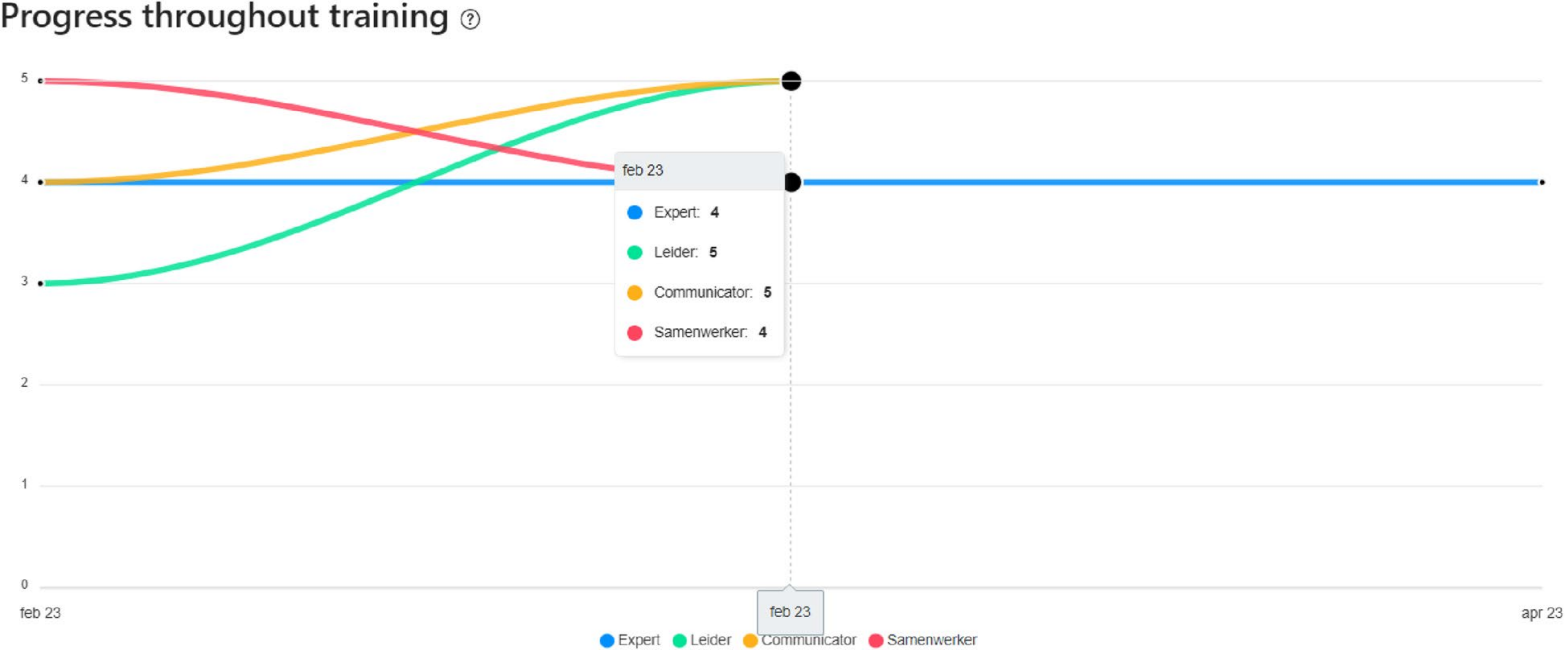
Example of a visualization of competency development in an ePortfolio, based on the CanMEDS roles

 After showing a possible visualization (Fig. 2), where the progress of the CanMEDS Roles is shown throughout a certain period of time, participants were enthusiastic and believed that this could add value to supporting continuity and identifying gaps.

“Something visual is certainly good, certainly mentors inquiring parties. But also students, when they can quickly consult their current competence level, what they have done, or what challenges there still are…” *(P8 – general practice)*.

### Example of a visualization of competency development in an ePortfolio, based on the CanMEDS roles

When asking participants about their ideal image of a visualization to support competency development during WIL, they expressed some requirements for the visualization of competency development that could benefit effective and continuous competency development.

First, participants believed that the viewing restrictions of the visualized competencies should depend on the stakeholder role e.g., student, mentor, or educator. For example, sometimes mentors might not need to see the complete content while an educator might want to see it all.

“If you are an internship coordinator, you can see a lot more than if you are a mentor of one specific internship.” *(P4 – pediatrics)*.

Second, participants prioritized the visualization of the **sco**re of the educator as this score was seen as the most objective and indicative in the context of this study. According to participants, this score was also the one the final decision relied on.

“If you want to see an evolution, you have to visualize the score of the educator as this score is more reliable and more indicative. With mentors, you see a lot of differences. Even with educators, differences are difficult to eliminate.” *(P10 – nursing)*.

Third, participants preferred visualizations of competencies at a higher level of the competency framework (e.g., at the level of the CanMEDS roles) and not at the level of key competencies, enabling competencies, or assessment criteria. Focusing on lower-level competencies or individual assessment criteria could be too concrete, and their number would hinder a structured visualization. Furthermore, visualizations at a higher level improves the visualization of progress and show a more global overview of a student’s competency development which is more important than the visualization of multiple separate assessment criteria.

“If you visualize at the level of assessment criteria or behavioral indicators, you will not be able to see growth. These are all small, separate parts of a larger competency. So I think you want to see growth at competency level and not at the level of assessment criteria. In this way, you can see if a student grows or not.” *(P14 – associate degree nursing)*.

### Requirements for competency development after graduation

In addition to monitoring competency development during students’ educational program, taking their competency development after graduation was a topic that was questioned. Participants highly valued the continuous competency development after graduation emphasizing the need for continuously develop competencies, also after graduation.

“We are thinking about that. The great thing would be if that could last their whole career. I would like that very much.” *(P13 – business management)*.

In line with the recommendations for each of the requirement types, several recommendations were provided by the participants in order to improve competency development. According to participants, the visualization of competency development can offer a support in the transition from bachelor to master, to support future employment, or to support lifelong learning. In the context of future *employment*, participants stated that visualizations might be helpful as a kind of curriculum vitae and/or to distinct yourself from others. However, participants also thought that graduates might be less motivated to take these visualizations with them because they already had their degree and/or job. Some participants stated that taking the visualizations of competency development after graduation might have benefits in the context of *certification*, or as a part of *lifelong learning*. However, our analysis revealed two barriers for CPD engagement in practice. The lack of intrinsic motivation, combined with the lack of a mandatory CPD program, partly due to the slow changes occurring in practice due to the policy, might hinder healthcare professionals to engage with CPD. Participants explicitly mentioned that when continuing education or certification would be an obligation, graduates would be more motivated to keep their competency development on track.

“You must be very disciplined if you hop from one job to another and keep track of your competency development. Because it is a dead end after 40 years… then the competencies and accompanying examples are not up to date anymore.” *(P13 – business management)*.

Moreover, the visualization of a student’s learning opportunities seemed interesting to participants in the context of employment or lifelong learning to see how experienced someone is with certain competencies.

“It would be interesting to see by visualizations just before graduation which students never had the opportunity to conduct a consultation. In this way, we could try to create learning opportunities to fill this gap.” *(P6 – speech therapy)*.

## Discussion

The study identified the content related to WIL which is required to achieve the essential competencies that healthcare students need in their future jobs and its relationship to CBE that focuses on daily practice, which brings into play the continuous development of competencies during WIL. The study revealed eight types of requirements. It is noteworthy that certain requirements were met in one healthare educational program while they were absent in another. This highlights the great differences in the form that CBE is taking in different healthcare educational programs and healthcare practices. The identified requirements encompassed various aspects, including:

1. *Competency frameworks*: The selection, utilization, and implementation of appropriate competency frameworks played a crucial role in competency development.
2. *Reflection and feedback*: Encouraging students to engage in reflective practices, also at a higher level than daily practices, and providing constructive feedback were identified as important factors in enhancing competency development.
3. *Assessment*: The design and implementation of robust assessment methods and tools were essential for evaluating competency development accurately and support its effectiveness and continuity.
4. *Continuity of development*: Ensuring the continuity and progression of competency development throughout a student’s educational career and thereafter emerged as a critical requirement.
5. *Mentor involvement*: Active involvement and guidance from mentors significantly influenced competency development during WIL.
6. *ePortfolios*: The use of ePortfolios provided a valuable digital tool for documenting and supporting competency development.
7. *Competency development visualizations*: Visual representations and tools that visually mapped competency development and progress facilitated the understanding and monitoring of competency development.
8. *Competency development after graduation*: Recognizing the importance of ongoing competency development even after graduation was identified as an essential requirement.

Significant variations in the extent to which these requirements were met across different educational programs and workplace settings were highlighted. These variations underscored the considerable differences that exist not only between educational programs but also among diverse workplace environments serving as learning contexts for students. Whether or not an educational program addresses the requirements or not might influence the implementation of CBE and a student’s effective and continuous competency development. To illustrate the importance of the identified differences between the educational programs and demonstrate possible consequences, some examples were outlined below:

- ○ The large diversity of competency frameworks might complicate WIL as mentors might be confronted with different forms and assessment tools. Moreover, the differences when it comes to predefined competencies might hinder interprofessional collaboration and communication. For example, when working with other healthcare professionals or giving peer-feedback.
- ○ Some stakeholders want to take along all information about the student (e.g., reflections, feedback forms, assessments, etc.) throughout the entire program while others prefer to restrict this content. First of all, restricting this content might hinder the continuity of (WIL) learning. Second, the discrepancy between different stakeholders might impede progress and innovation when it comes to curriculum design, adapted to the needs of the patient and the healthcare system.

### Requirements for competency frameworks

The significant disparities observed between educational programs were reflected in the diverse range of competency frameworks utilized. Notably, no two educational programs employed identical competency frameworks, posing challenges for workplaces in understanding and effectively supporting students’ competency development. While various competency frameworks exist, it became evident that the high degree of context-specificity within each educational program made it difficult to find an existing framework that perfectly aligned with its specific needs. This observation aligns with the findings of Janssens et al. (2022), whose study validated the CanMEDS competency framework at the level of key competencies in order to establish a standardized framework across different healthcare educational programs. The study also acknowledged the substantial context-specificity present. However, the CanMEDS competency framework demonstrated applicability to many educational programs when implemented at a higher level (e.g., CanMEDS roles or key competencies) rather than focusing on enabling competencies or assessment criteria (also called behavioral indicators) [12].

The content of the competency framework was multi-layered when looking at current practices. Our results revealed that the diversity in number of competencies but also the levels within the framework caused problems. Accordingly, literature already stated that the development and implementation of a competency framework might be complex due to the ambiguity and vagueness of the used competency framework [36]. If the levels of a competency framework were unclear, participants thought that adding a rubric to the framework might clarify expected competence levels and optimize continuous competency development. Previous studies confirmed the value of a rubric, in assessing but also in teaching when it is formatively used. However, it seemed essential that the descriptions within the used rubric are concrete and context-specific but in practice, this was often not the case [35].

### Requirements for reflection and feedback

Our study revealed that the continuity of competency development could be at stake. One reason that popped up was the disagreement between participants about taking reflections and feedback of students to consecutive internships. The majority of the participants was enthusiastic about the taking along students’ competency development. But when it came to taking all reflections and feedback throughout the educational program, they doubted. Most of the participants preferred only a summary of the reflections and feedback and not the full content. However, previous research underscored the importance of continuity within the learning process [37]. In contrast, another study revealed that reflection and feedback were often neglected while the focus on assessment was high which might hinder continuous competency development [38]. Taking the findings of our study and the importance of continuous competency development into account, it might be an option to refrain from aiming at detailed reports and feedback and to prioritize the visualization of competency development. The challenge remains in finding a balance between too little and too much information to take along during a student’s entire educational career.

### Requirements for assessment

Some participants in our study did not support the fact that all assessment forms were taken along the complete educational program as they thought students should start with a clean slay. This might hinder the continuity of competency development. However, the recent introduction of programmatic assessment emphasizes the importance of a continuous and dynamic approach when it comes to assessment. Programmatic assessment is defined as ‘an assessment system which comprises of low, mid and high stakes assessments conducted throughout the program year’ [38]. It enables longitudinal assessment that not only points to assessment *of* learning but also facilitates assessment *for* learning, optimizing the continuity of competency development [39, 40]. Without this continuous, longitudinal approach of assessment, the implementation of CBE might remain insufficient, focusing on the assessment *of* learning [38, 41]. Future research could zoom in on the possible reasons for the reluctance of educators and mentors when it comes to taking competency development along; and on possibilities to resolve it.

### Requirements for the continuity of competency development

Our study revealed multiple requirements in relation to the aspect of continuity. The educational system, the use of the same competency framework, the visibility of the content of reflections and assessment, the available learning opportunities, and the diverse learning environments influence the continuity of students’ competency development. Our findings showed that requirements in relation to these topics were often not met due to the rigidity of the educational system, the diverse competency frameworks, the reluctance of stakeholders, the limited number of learning opportunities, and the various, non-comparable learning opportunities within diverse learning environments. This might imply that the programmatic assessment approach as introduced by Van der Vleuten [38] is still ineffectively implemented as the focus of programmatic assessment lies within the longitudinal continuity aspect. This is in accordance with previous research stating that the continuity of competency development should be emphasized more [39, 40].

Furthermore, our findings revealed that the transfer of the theoretical competency framework into practice was problematic, causing challenges in relation to the continuity of developing competencies. When competency development was not completely embedded in the wider curriculum, the responsibility lied with the student’s educators according to our participants. This might hinder continuous competency development due to the subjectivity and emphasis on personal differences. This is in accordance with earlier research stating that the needed competencies and their development need to be thoroughly integrated into the curriculum so that a holistic picture of competency development can be provided and continuity can be ensured [28, 42]. Another study showed that it seemed difficult for nurses to apply in practice what they have learned in nursing schools [43]. They also stated that the inappropriate curricula, focusing on technical competencies, might further complicate continuous competency development which is in alignment with our findings [43].

In the literature, there are plenty of studies investigating programmatic assessment [40, 44]. However, although there are a few studies investigating the aspect of continuity besides assessment, especially in function of learning (e.g., taking reflection and feedback along through the program) but the evidence is limited [45]. After graduation, there are some studies focusing on the transition from being a student to being a professional. For example, a recent study focused on this gap between education and practice but as in most of these studies, the focus was on facilitating the transition in relation to performance and well-being, and not on the further development of competencies [46].

### Requirements for mentor involvement

From our results arose that the optimal scenario of mentor involvement was impossible in practice. The one-to-one guidance that was preferred by participants was not doable due to the lack of healthcare professionals and the high workload. In the literature, the benefits of faculty development (= providing professional development training to faculty members) has been introduced a long time ago [47]. In addition, the importance of a student sharing all feedback and assessment information with one well-educated mentor was underscored. However, the literature about how this happens or should happen in practice is scarce [38].

### Requirements for ePortfolios

Our results revealed that, although an ePortfolio was used to support continuity, competency development often remained fragmented. This can be caused by the focus on daily practice and individual competencies; and the lack of focus on the continuity of competency development. This is in accordance to previous studies stating that students perceived an ePortfolio as a fairly accurate, but fragmented picture of a student’s development at the workplace. They stated that an ePortfolio rather provided snapshots than a complete picture of a student’s continuous competency development mostly containing descriptions of daily performances and focusing on technical competencies [30].

The availability of an ePortfolio, independent of time and location was perceived as a requirement in our study. These results align with the literature stating that the unavailability of an ePortfolio might hinder effective CBE implementation and thus competency development [48].

### Requirements for competency development visualizations

The opportunity to use visualizations or visual tools, e.g., in an ePortfolio, to support competency development might offer advantages according to our participants. There is a limited amount of literature investigating the added value of visualizations of competencies to support competency development. One study emphasized the value of using an ePortfolio with its integrated visualizations to support assessment and competency development. However, there were no concrete suggestions for these visualizations within this study [40]. Future research might examine the effect of using an ePortfolio with visualized competencies and competency development on learners’ (perceived) learning process and outcomes.

### Requirements for competency development after graduation

Although our findings revealed that carrying competency development forward after graduation might be interesting, the effective implementation in practice remained complex. Earlier research stated that the competencies of the learner must be made explicit during the complete educational program, during the transition from one program to another, and during the transition into practice. They stated that the development of a competency-based model for CPD may provide a base for developing a new vision of CPD [49]. Our study showed that the intrinsic motivation of graduates to learn after graduation needs to be high to succeed in doing so. Participants mentioned that when continuing education or certification would be an obligation, graduates would be more motivated to keep their competency development on track. However, to the best of our knowledge, there is no research confirming these statements. However, previous research did show that the attitude and commitment to the nursing profession were essential for healthcare professionals’ competency development [43].

In our study, only one stakeholder group, namely educators/internship coordinators, provided their insights on how the continuity of competency development could be optimized and effective competency development could be attained. Future research might focus on the perspectives of mentors and students on continuous competency development, before and after graduation.

## Conclusion

This study aimed to examine how a student’s competency development during WIL in healthcare education can be optimized. The participants identified eight types of requirements that might contribute to the optimization of competency development during WIL in healthcare education, namely requirements in the context of (1) competency frameworks, (2) reflection and feedback, (3) assessment, (4) the continuity of competency development, (5) mentor involvement, (6) ePortfolios, (7) competency development visualizations, and (8) competency development after graduation.

The identification of requirements might serve as a first step to improve the competency development of students, both in terms of effectiveness and continuity. However, the multiple requirements not being met, the large differences between educational programs, and the high complexity of current learning contexts might complicate a student’s competency development. As a result, it can be stated that that both the healthcare workplaces and the educational institutions are not ready (1) to provide a continuous, tailor-made curriculum for each student to optimally support effective and continuous competency development, nor (2) to implement a lifelong learning component to guarantee continuous competency development even after graduation.

### Implications for practice

The identifications of requirements for competency development during WIL in healthcare education might help to optimize curriculum design and the collaboration between the educational institutions and healthcare workplaces. Zooming in on the first aspect of curriculum design, two key recommendations were established.

First, a uniform competency framework might offer opportunities without losing sight of the context-specificity for example, the integration of context-specific behavioral indicators into a comprehensive competency framework or adding context-specific descriptions to a more general rubric. For educational institutions and students, this might be beneficial for learning and working together for example, by providing and receiving peer feedback. Moreover, mentors at the workplace might encounter a limited variety of competency frameworks enabling an easier-follow up of students including a more efficient assessment process.

To take along competency development throughout the educational program and to emphasize formative assessment, a programmatic assessment approach might be implemented. The reluctance of mentors and/or educators for taking along reflections, feedback, and assessments might cause problems for doing this. However, increasing the involvement of these parties might be a possible solution for example, when developing and implementing an ePortfolio to support the continuity of competency development.

To optimize competency development after graduation, in the context of CPD, the motivation of graduates needs to be increased. An increased emphasis on the need of CPD should already be mentioned at the start of the educational program. Discussing the possibilities of CPD, showing the ways in which they can engage with CPD, and make CPD doable in time and resources while monitoring the work-life balance are things to do for educational institutions as well as for healthcare workplaces and policy. In this way, the internal motivation of graduates could be increased.

### Limitations

First, this study was conducted based on a well-defined and strictly conducted methodology, taking into account the heterogeneity of the involved programs. However, not every educational program was represented in the sample, and a purposive sampling approach was used due to practical considerations. This may lead to certain results differing from those obtained when we would have included other educational programs. However, since the perspectives of the involved stakeholders from various educational programs on most issues are similar (e.g., opinions on CPD, views on the continuity of the learning process, opinions on decisive factors in assessment, etc.), we can assume that the results would not deviate significantly.

Second, the diverse nature of the involved programs can be seen as a strength, as it brings together different viewpoints and allows for the identification of variations. However, it can also be viewed as a weakness because the differences may be too significant to generalize the results.

Third, although the researchers conducting the interviews were highly capable and had equivalent knowledge on the topics under discussion, the presence of different interviewers might introduce bias to the results. Nevertheless, the coding process was carried out by two researchers, and codes and themes were discussed multiple times within the research team.

### Supplementary Information

**Supplementary Material 1.**

## Data Availability

A Data Management Plan was constructed via https://dmponline.be and monitors the storage and access to the data (ID: 107491). Data are available on request by contacting the main author (Oona.Janssens@UGent.be).

## Abbreviations

WIL: Work-integrated learning
CBE: Competency-based education
CanMEDS: Canadian Medical Education Directives for Specialists
ACGME: Accreditation Council for Graduate Medical Education
CPD: Continuous professional development
EPA: Entrustable professional activity
