# Supplementary material for: The identification of requirements for competency development during work-integrated learning in healthcare education
Source: BMC Med Educ. 2024 Apr 22;24:427. doi: 10.1186/s12909-024-05428-9 (PMC11034030; doi:10.1186/s12909-024-05428-9)
Supplement: Supplementary file 1 — Supplementary Material 1. [file 12909_2024_5428_MOESM1_ESM.docx]

# Supplementary Info

# Appendix 1: topic guide

**TOPIC GUIDE**

Implementation of competency frameworks

1. Which **competency framework** do you use within the educational program you’re part of?
2. Does this competency framework remains the same for **each program year**?
3. Is the **workplace aware** of the used competency framework when a student is on an internship?
4. Does this competency framework consist of concrete **behavioral indicators**?
5. At **what level** do you want this competency framework to be **implemented** during internships?

Facilitators and barriers for competency development

1. Is competency development of students **visualized**?
2. **Would you like** competency development to be **visualized**?
3. What is your **ideal image of the visualization** of competency development?
4. Would you like that competency development is captured **throughout the entire program** or rather keep these for separate internships or program years?
5. Which **stakeholders** (e.g., students, mentors, educators) would you like to be able to consult this **competency growth**?
6. Would you like to take this competency development with you **after graduation**?

# Appendix 2: codebook

The first column shows the themes while the second column presents the sub-themes. The overarching concepts of requirements were shown above the related themes and sub-themes.

| REQUIREMENTS FOR COMPETENCY DEVELOPMENT | |
| --- | --- |
| *ASSESSMENT REQUIREMENTS* | |
| (No) discrepancy between score of student and score of mentor | If scores of student and mentor match, you can have deeper assessment conversations |
|  | Mismatch between student and mentor |
| Assessment criteria requirements | Availability of behavioral indicators or assessment criteria |
|  | Behavioral indicators or assessment criteria need to be very clear for the workplace in the context of dual learning |
|  | Concretizing generic competencies asks a lot of effort |
|  | Criteria are further explained |
|  | Explanation for each assessment criterium helps to reduce the number of criteria |
|  | Limited number of sub-levels provide less concrete assessment criteria |
|  | Workplace thinks behavioral indicators or assessment criteria have to little link with the workplace |
|  | Workplace thinks the formulation of indicators or criteria is too complex |
| Assessment is often subjective | Educational program based on gut feeling, not on pedagogical theories |
|  | Uncertainties about certain students are being discussed during intercollegiate conversations |
| Characteristics of final score of internship | Final score is often a compromise |
|  | Giving final score happens manually |
|  | Interpretation of final score differs between educators |
|  | Not every score contains the same content |
| Educator role | Educator gives final score |
|  | Educator has no direct role during the assessment |
|  | Educators should have the responsibility to decide whether or not a skill is achieved |
| Same assessment instrument for bachelor and master medicine |  |
| Scale is good for assessment of separate competencies but a global score is needed |  |
| Score might be necessary next to a colors scale |  |
| Score should be clear |  |
| Scores below expectations need to be discussed but that is not easy |  |
| Scores of mentors differ a lot |  |
| Scores should be added to the spider diagram |  |
| Scoring happens outside ePortfolio |  |
| Scoring is based on student and mentor |  |
| Scoring methods | Color codes or color scale |
|  | No score, but color code |
|  | Giving a score is not necessary for lower level competencies |
|  | Higher level competencies need to achieved, not lower level competencies |
|  | Level of scoring |
|  | Minimum criteria to be met |
|  | Only the lowest level of competencies is used in the ePortfolio because only this level is assessed |
|  | Pass-fail |
|  | Differentiation is not necessary, pass is sufficient |
|  | No score, only pass-fail |
|  | Pass fail can't be used at the educational institution |
|  | Pass fail scale is more clear |
|  | Pass fail scoring method |
|  | Pass or fail are concretely described depending on assessment criteria |
|  | Pass or fail can not be based on (opportunistic) exposure |
|  | Qualitative end assessment |
|  | You have to achieve a complete role before you can become a good professional |
|  | Scoring on higher level of competencies only when it adds value for the student |
|  | Scoring on higher level, not criteria |
| Scoring persons | Conversation about competency development between educator, mentor and student |
|  | Student will score more in the middle |
|  | Students don't score themselves |
|  | Students score themselves |
|  | Selecting artefacts with best performance can be a problem with students |
|  | Student scores himself or herself at the start of the program |
| Time investment of assessment is important |  |
| *COMPETENCY FRAMEWORKS REQUIREMENTS* | |
| A combination of different competency frameworks is ideal |  |
| CanMEDS competency framework | Adapted CanMEDS competency framework is used |
|  | CanMEDS competency framework can be beneficial to use because of the uniformity between educational programs |
|  | CanMEDS competency framework has a strong link with practice |
|  | CanMEDS competency framework is supplemented with professional activities as a lower level |
|  | CanMEDS is interesting but shows a lot of overlap |
|  | CanMEDS is not known |
|  | CanMEDS is used as a framework but not in practice |
|  | CanMEDS role are more or less important depending on the internship |
|  | Level names within CanMEDS |
| Competency framework development | Assigning existing competencies to EPA's |
|  | Assigning explanation to competency |
| Competency framework needs to be context- and time-specific | Assessment instrument differs per internship but competency framework remains the same |
|  | Assessment instrument is longer at later internships |
|  | Certain EPAs are important in one internship and in another not |
|  | Competency framework is adjusted through the years, based on expected competency development |
|  | Competency framework needs to be adjusted to the workplace |
|  | Competency framework starts from EPA approach |
|  | Expectations per program year are more motivating then holding on to the expectations for graduations |
|  | Expert role not applicable in the first program year |
|  | Level of competence needs to depend on program year |
| Making EPAs not visible in ePortfolio when they are not applicable |  |
| Only competencies or EPAs that were selected at the start can be assessed at the end |  |
| Experience with and expectations of EPAs | Choice of EPAs based on stronger link with workplace |
|  | Coupling artefacts to EPAs |
|  | EPAs are more difficult to see as a tree structure |
|  | EPAs offer opportunities |
|  | EPAs_benefits and challenges |
|  | Evaluation of formulated learning goals based on EPAs at the end of an internship |
|  | Expected switch to EPA within a year |
|  | Experience with EPAs |
|  | Formulating learning goals based on EPAs at the start of an internship |
|  | No experience with EPAs |
| Generic competencies vs. technical competencies |  |
|  | Emphasizing the need to perform a certain skill might cause an emphasis on technical competencies |
|  | Focus on technical competencies |
|  | Generic competencies are at least as important as technical competencies at the workplace |
|  | Generic competencies remain the same during all internships |
|  | Generic competency development is easier through the years than technical competency development |
| Growth structure or rubric | Competency development is more clear because the competency framework consists of a growth structure (EVA) |
|  | Rubric defines explanations for each assessment criterium |
| Levels of competency framework need to be determined | Balance between too many sub-levels and too little sub-levels |
|  | Workplace is happy with two level competency framework |
|  | Levels within EPAs |
|  | Three levels of competencies is ideal |
|  | Too many sub-levels provide too much assessment criteria |
|  | Too much levels to score and visualize complicate the learning process |
| No distinction between knowledge and skills anymore |  |
| Number of competencies | Number of lists and competencies needs to be limited |
| Same competency framework over years | Opportunities to use a uniform competency framework from bachelor to master |
|  | Same competency framework is used through the program years but the level to attain is adjusted |
|  | Same competency framework through all program years |
| Transferring competencies into practice |  |
| Weighting competencies | All competencies have the same weight |
|  | Competencies besides the minimum competencies have the same weight |
|  | Some competencies have more weight than others |
|  | Weight of competencies happens manually |
|  | Weight of competencies in algorithm |
| Workplace awareness and knowledge | Students and educators are not aware of competency framework |
|  | Training the workplace about competency frameworks |
| *EPORTFOLIO REQUIREMENTS* | |
| Adjustable week planning in ePortfolio |  |
| All necessary features need to be in one ePortfolio |  |
| Archiving artefacts is a nice feature |  |
| Automation appeals | Automatic input of competency framework in ePortfolio |
|  | Automatic pass fail in ePortfolio |
|  | Automatic score after assessment |
|  | Filtering competencies based on the used competency framework |
|  | Filtering visualizations of competency development through an internship, a semester, a year, etc. |
|  | Notification that feedback is given |
|  | Notification that feedback of mentor is needed in ePortfolio |
|  | Possibility to receive an e-mail as notification |
| Availability of device for using ePortfolio is important | Smartphone as ePortfolio device offers added values |
| Benefits of ePortfolio over paper-based portfolio |  |
| Capturing learning moments or artefacts and feedback on these learning moments or artefacts |  |
| Concepts used in ePortfolio are essential | Level names within the ePortfolio |
|  | The concept competency is not used anymore |
|  | The concept of artefact might be unclear |
|  | Word choice of features in ePortfolio |
| CV or report in ePortfolio | A CV out of an ePortfolio might be interesting when you can filter for certain competencies so that you can proof your experience |
|  | Pulling a CV out of the ePortfolio might be interesting when it is generic |
|  | Pulling reports out of the ePortfolio is an interesting feature |
| Detecting competency gaps through ePortfolio |  |
| ePortfolio can be adjusted by educational programs |  |
| ePortfolio needs to be simple | Competency framework in ePortfolio needs to be simple |
|  | It should be possible to use the ePortfolio easily at the workplace, at the bus, etc. |
|  | Simplicity of ePortfolio is good |
| Goal of ePortfolio is to support and visualize competency development |  |
| Has to be clear on what type of artefacts feedback is needed |  |
| Higher level competency is automatically scored when lower level competency is scored |  |
| Initiating the validation of feedback by the mentor or educator could be interesting |  |
| Involving mentors in ePortfolio is innovative | Coupling workplace to student in ePortfolio |
| Large uploading opportunities | Uploading multimedia offers opportunities |
|  | Uploading shared files in ePortfolio |
|  | Uploading video in ePortfolio is seen as beneficial |
| Learning goals should be clear in ePortfolio |  |
| Optimizing the structure of the learning process by an ePortfolio |  |
| Overview of artefacts when clicking on visualization of certain competency would be beneficial |  |
| Quality is more important than quantity in ePortfolio |  |
| Registration of hours of work needs to be in ePortfolio |  |
| Skills list in ePortfolio |  |
| Skills list needs to be taken to future internships so digitalization is necessary |  |
| Styling options (e.g. bold) are beneficial |  |
| Time investment of ePortfolio is important |  |
| Timetable of working hours can be more flexible through ePortfolios |  |
| Use of ePortfolio besides during workplace learning |  |
| Using ePortfolio to evidence other competencies than competencies at the workplace |  |
| Visibility of ePortfolio content depending on role of ePortfolio user |  |
| Wanting a learning agenda |  |
| *REQUIREMENTS FOR REFLECTION AND FEEDBACK* | |
| Reflections about complete program or global competence are scarce |  |
| *REQUIREMENTS FOR COMPETENCY DEVELOPMENT AFTER GRADUATION* | |
| Having your degree makes visualization after graduation less useful |  |
| Pulling out a CV or PDF out of the ePortfolio as a start after graduation might be interesting |  |
| Taking an ePortfolio after graduation requires discipline |  |
| Visualization after graduation based on experience and not on scores |  |
| Visualization after graduation can be beneficial when you are changing jobs |  |
| Visualization after graduation for certification |  |
| Visualization after graduation for LLL |  |
| Visualization after graduation might help to distinct yourself from others |  |
| Visualizations after graduation will not be used because LLL after graduation is not yet obligated |  |
| When visualizations are taken after graduation, the ePortfolio should fit to each company |  |
| *REQUIREMENTS FOR CONTINUITY OF COMPETENCY DEVELOPMENT* | |
| Competency development differs between different students |  |
|  | Competency development depends on individual student |
|  | Little students score above expectations because it is already a strictly selected group |
|  | Not every student can reach the same program year goals |
|  | Not every students starts from zero |
| Competency development through internships is difficult because of limited internship places |  |
| Competency development through internships is difficult because of the different contexts |  |
| Current situation is not ready for tailor-made curriculum of students |  |
| Discussing competency development after three months of internship |  |
| Each internship should be a clean slay |  |
| Learning goals are formulated based on previous internships but scores of previous internships are not taken |  |
| Learning process needs to be ongoing |  |
| Mentors only see students progress during current internship |  |
| Openness of students when scores are taken to future internships |  |
| Preferences about competency development through internships |  |
| Problems for competency development due to students' responsibilities |  |
| Student can choose who might consult his ePortfolio |  |
| Student can make errors in coupling artefacts to competencies |  |
| Students determine whether mentors can see the content of their ePortfolio |  |
| Students might think that they don't have to develop competencies further if they are visualized as sufficient |  |
| Self-assessment of students is not preserved |  |
| Taking artefacts and feedback through internships should not be possible at all time |  |
| Visibility of competencies ends at the end of a program year |  |
| *REQUIREMENTS FOR MENTOR INVOLVEMENT* | |
| Coupling mentors to students | Assigning mentors to students |
|  | Coupling one mentor to one student is not possible (anymore) |
|  | Coupling only one mentor to a student |
| One-to-one guidance at the workplace |  |
| *REQUIREMENTS FOR COMPETENCY DEVELOPMENT VISUALIZATIONS* | |
| If students perform on expected level, you will not need to see growth in visualizations |  |
| Varying line diagram does not evidence less competence |  |
| Visualization is seen as difficult because of the use of a rubric assessment approach |  |
| Visualization of competencies might be difficult because competencies have other weights |  |
| Visualization of global competence is difficult |  |
| Visualization of number of artefacts does not show the competence |  |
| Visualization of too much competencies is not achievable |  |
| Visualizations can be demotivating |  |
| Benefits of line diagram to see progress |  |
| Benefits of spider diagram of individual competencies |  |
| Boxplot of competencies could offer opportunities |  |
| Consulting competency development through visualization |  |
| Direct visibility of student's shortcoming competencies |  |
| Link between artefact and competency development (visualization) |  |
| Overview or visualization of scores of student and mentor is interesting |  |
| Overview or visualization of scores of student, mentor and educator |  |
| Possibility to consult visualization of competency growth should be available at any moment |  |
| Spider diagram is interesting to detect competency gaps |  |
| Use of colors | Color codes ensures that competency development can easily be captured and consulted |
|  | Visualizations with colors are clear |
|  | Workplace likes the color codes |
| Visualization of competency development is more important than snapshots of performance |  |
| Visualization of competency profile e.g., spider diagram could offer opportunities after graduation |  |
| Visualization of exposure | Spider diagram shows experience with competencies and not mastering |
|  | Visualization of competencies students already dealt with |
|  | Visualization of competencies that students worked on is easier than visualization of competency development |
| Visualization of growth per competency | Visualization per competency per internship |
| Visualization of number of artefacts could be beneficial for gap detection |  |
| Visualization preferences | Level of visualization |
|  | Objective visualization of competency development by taking the same test over and over |
|  | Score of educator is preferred to be visualized because of the higher reliability |
|  | Visualization based on red or green, no in between |
| Visualizations appeal to me |  |
| Visualizations can help to have a quick overview of outlying competencies |  |
| Visualizations need to be easy and need to ease the learning process |  |
| Visualizations of competency development can help to detect competency gaps |  |
| Visualizations taken through the years | It might be interesting that mentors see the visualization of competency development of previous internships |
|  | Taking visualizations through internships can be difficult because the content is open to interpretation |
|  | Taking visualizations through internships or program years can be difficult because of the educational system that needs to close after a program year |
|  | Taking visualizations through the program year is nice to have but no need to have |
|  | Visibility of visualizations through internships for educators |
|  | Visualizations of program year goals |
|  | Visualizations taken from bachelor to master |
|  | Visualizations taken through internships or program years |
